# *SOX2* participates in spermatogenesis of Zhikong scallop *Chlamys farreri*

**DOI:** 10.1038/s41598-018-35983-3

**Published:** 2019-01-11

**Authors:** Shaoshuai Liang, Danwen Liu, Xixi Li, Maokai Wei, Xiaohan Yu, Qi Li, Huixin Ma, Zhifeng Zhang, Zhenkui Qin

**Affiliations:** 1 Ministry of Education Key Laboratory of Marine Genetics and Breeding, College of Marine Life Sciences, Ocean University of China, Qingdao, 266003 China; 2 The Institute of Oceanology, Chinese Academy of Sciences, Qingdao, 266000 China

## Abstract

As an important transcription factor, SOX2 involves in embryogenesis, maintenance of stem cells and proliferation of primordial germ cell (PGC). However, little was known about its function in mature gonads. Herein, we investigated the *SOX2* gene profiles in testis of scallop, *Chlamys farreri*. The level of *C*. *farreri SOX2* (*Cf-SOX2*) mRNA increased gradually along with gonadal development and reached the peak at mature stage, and was located in all germ cells, including spermatogonia, spermatocytes, spermatids and spermatozoa. Knockdown of *Cf-SOX2* using RNAi leaded to a mass of germ cells lost, and only a few spermatogonia retained in the nearly empty testicular acini after 21 days. TUNEL assay showed that apoptosis occurred in spermatocytes. Furthermore, transcriptome profiles of the testes were compared between *Cf-SOX2* knockdown and normal scallops, 131,340 unigenes were obtained and 2,067 differential expression genes (DEGs) were identified. GO and KEGG analysis showed that most DEGs were related to cell apoptosis (*casp2*, *casp3*, *casp8*), cell proliferation (*samd9*, *crebzf*, *iqsec1*) and spermatogenesis (*htt*, *tusc3*, *zmynd10*, *nipbl*, *mfge8*), and enriched in p53, TNF and apoptosis pathways. Our study revealed *Cf-SOX2* is essential in spermatogenesis and testis development of *C*. *farreri* and provided important clues for better understanding of *SOX2* regulatory mechanisms in bivalve testis.

## Introduction

SOX2 is a member of the SOX (SRY-related HMG-box) family with a high-mobility-group (HMG) DNA-binding domain. Several studies have determined that SOX2, as an important transcription factor, participates in development processes of vertebrates, such as embryogenesis^1,2^, maintenance of stem cells^3–5^, neurogenesis^6–8^ etc. Recent investigations have also emerged that SOX2 is one of the transcription factors underlying the derivation of induced pluripotent stem cells^9–11^.

The role of SOX2 in gametogenesis has only been reported in mouse *Mus musculus*. Campolo *et al*.^12^ found that SOX2 participates in the proliferation of primordial germ cells (PGCs). However, the role of SOX2 in gametogenesis of mature adult of vertebrates and invertebrates has not been reported. Up to now, studies about *SOX2* related to gonad development are limited. Researchers found that *SOX2* mRNA is ubiquitously expressed in all tissues in *Hyriopsis schlegelii* and the expression level in the testis is higher than that in ovary^13^; besides, *SOX2* expression in testis raised gradually along with its development^14^, which could be divided into three stages: initiation of testis formation (4–11 months), a stable growth phase (12–24 months), and a reproductive cell development phase (25–36 months), indicating its role in the development of testis^13^. In *Paralichthys olivaceus*, *SOX2* transcripts is expressed in gonadal tissues and the transcript abundance in ovary is higher than that in testis^15^. Patra *et al*.^16^ found that, in rohu carp *Labeo rohita*, *SOX2* mRNA is expressed in various organs, as well as in the cultured proliferating spermatogonial stem cells (SSCs). The *SOX2* expression level is highest in SSCs and its transcript abundance is higher in testis than that in ovary. Moreover, SOX2 protein is presented in SSCs of *L*. *rohita*, suggesting the participation of *SOX2* in male germ cell development and stem cells maintenance of fish^16^.

Spermatogenesis is a complex process and lots of genes have been discovered involving in this process, such as *stra8*, *nanos3*, *ddx5*, *tspy1*, *ngn3*, etc.^17,18^. However, most of these researches were performed in vertebrates or model organisms, while in mollusks, the related studies are limited. As for bivalves, several spermatogenesis related genes (*adad1*, *shippo1*, etc.) were screened out through pyrosequencing in giant lion’s paw *Nodipecten subnodosus*^19^. In Zhikong scallop *Chlamys farreri*, multiple genes have been cloned and proposed playing roles in gametogenesis based on their expression pattern (*DAX1*, *β-Catenin* and *FOXL2*)^20–22^, while recently *KLF4* and *PIWI1* genes were validated involving in spermatogenesis and gametogenesis, respectively, through RNAi^23,24^. Besides, function of many *sox* family genes in gametogenesis have been identified and studied, such as *SOX2*, *SOX7*, *SOX 8*, *SOX 14* in pacific oyster *Crassostrea gigas*^25^; *SOX9* in pearl oyster *Pinctada margaritifera*^26^; *SOXE*, *SOXH* in Yesso scallop *Patinopecten yessoensis*^27^; *SOX2* and *SOXB2* in *C*. *farreri*^28,29^ and *SOX11* in *Pinctada martensii*^30^. However, the studies of these *SOX* genes are confined to the expression analysis. In this study, Zhikong scallop *C*. *farreri*, an important commercial marine bivalve in China, was employed and the abundance and cyto-location of *C*. *farreri SOX2* (*Cf-SOX2*) mRNA in testis were revealed by qRT-PCR and *in situ* hybridization. We further determined that *Cf-SOX2* participated in the regulation of *C*. *farreri* spermatogenesis and testis development by means of RNA interference (RNAi). Moreover, we performed transcriptome analysis on testes before and after *Cf-SOX2* knockdown and investigated expression profiles of *SOX2*-related genes. Our data provide important clues for better understanding of the molecular mechanism about spermatogenesis and testis development in bivalve mollusks.

## Results

### Temporal expression of *Cf-SOX2* in testes during the reproductive cycle

**Figure 1:**
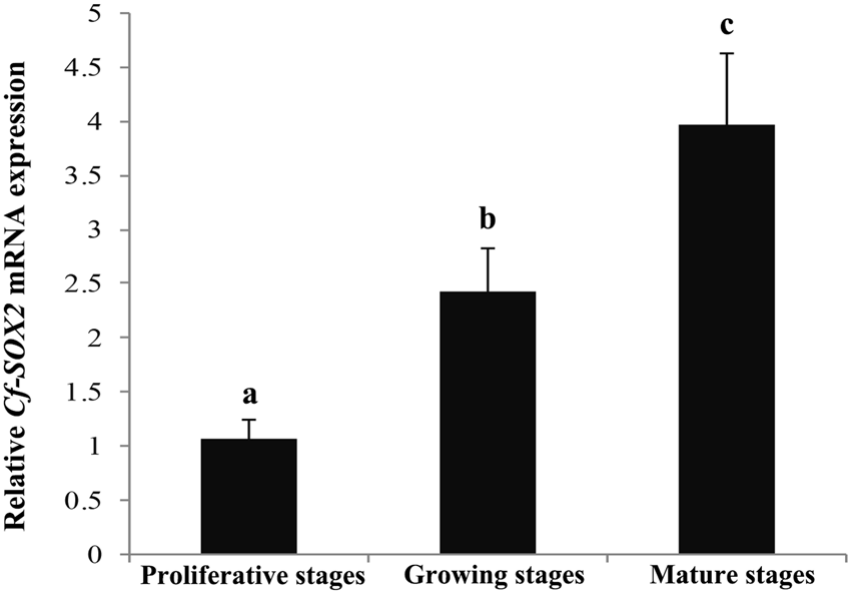
Relative expression levels of *Cf-SOX2* mRNA in *C*. *farreri* testes detected by qRT-PCR. Values are the mean ± SEM; n = 5. The expression level in the testis at proliferative stage is set as 1.00 to calibrate the relative expression at other stages. Different letters (a–c) indicate statistically significant differences (*P* < 0.05).

The relative levels of *Cf-SOX2* mRNA in testes were examined by qRT-PCR (Fig. 1). Abundance of the *Cf-SOX2* transcript increased significantly (*P* < 0.05) from proliferative stage to mature stage. And the expression levels were 2-fold and 4-fold higher in growing stage and mature stage than that in proliferative stage, respectively.

### Cyto-location of *Cf-SOX2* mRNA in testis during spermatogenesis

**Figure 2:**
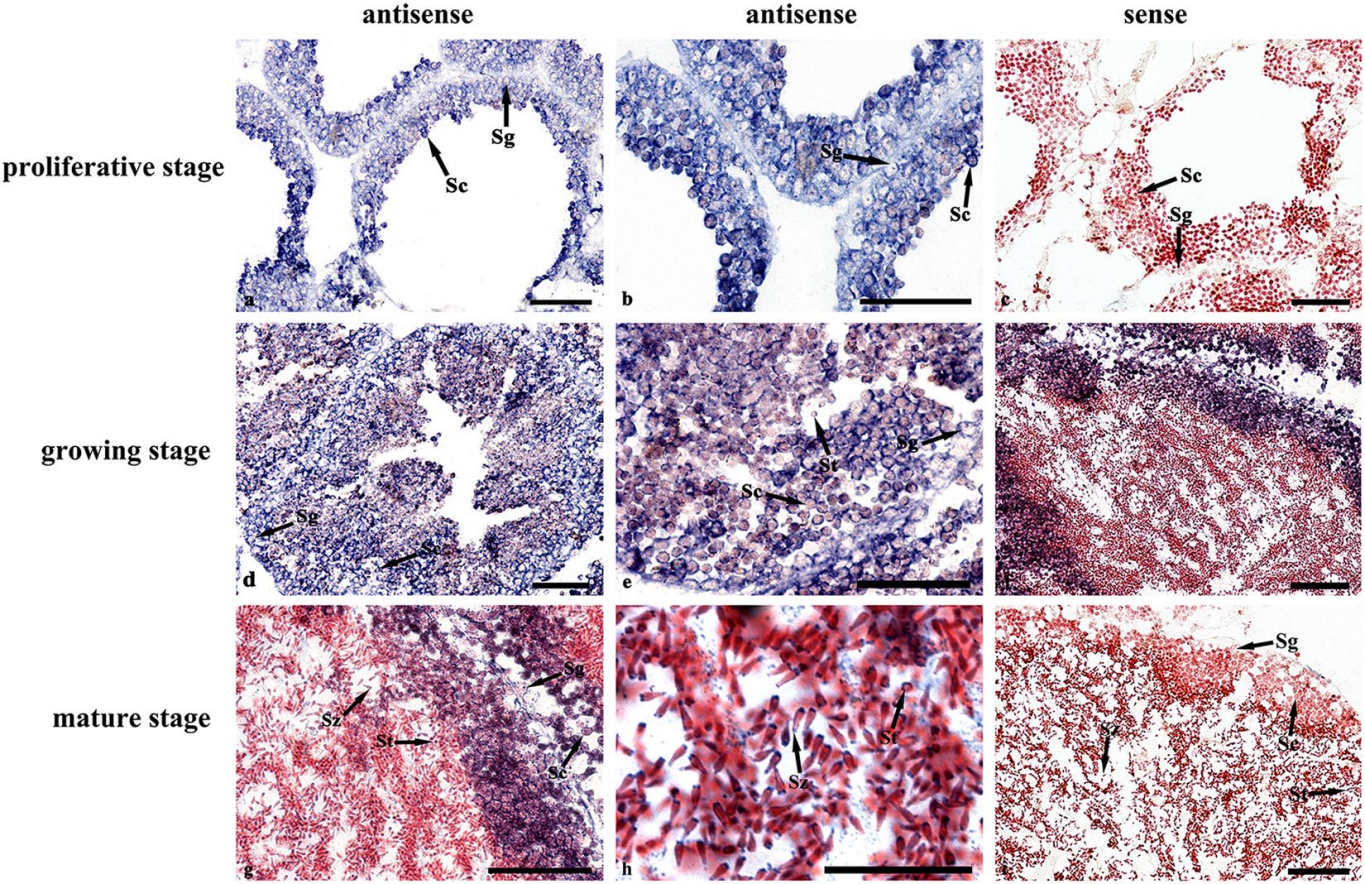
Cyto-location of *Cf-SOX2* mRNA in testes of *C*. *farreri* during spermatogenesis demonstrated by *in situ* hybridization. Positive signals with an antisense probe are indicated in blue (**a**,**b** and **d**–**h**). The negative control is conducted with sense probe (**c** and **i**). (**a**–**c**), Proliferative stage; (**d–e**), growing stage; (**f–i**), mature stage. Sg, spermatogonium; Sc, spermatocyte; St, spermatid; Sz, spermatozoon. Scale bar, 25 μm for h and 50 μm for others.

In *C*. *farreri* testis, spermatogonia, spermatocytes, spermatids and spermatozoa successively arranged from follicle wall to central region of germinal acini. The spatial location of *Cf-SOX2* mRNA in testes at different stages was demonstrated using *in situ* hybridization (Fig. 2). The *Cf-SOX2* transcripts were located in all germ cells, while intensity of the positive hybridization signals in spermatocytes was slightly higher than that in spermatogonia, spermatids and spermatozoa (Fig. 2a,b,d–f,g–h). Moreover, no hybridization signal was detected in testes with the sense probe (Fig. 2c,i).

### *SOX2* knockdown led to a testis developmental retardance in *C*. *farreri*

**Figure 3:**
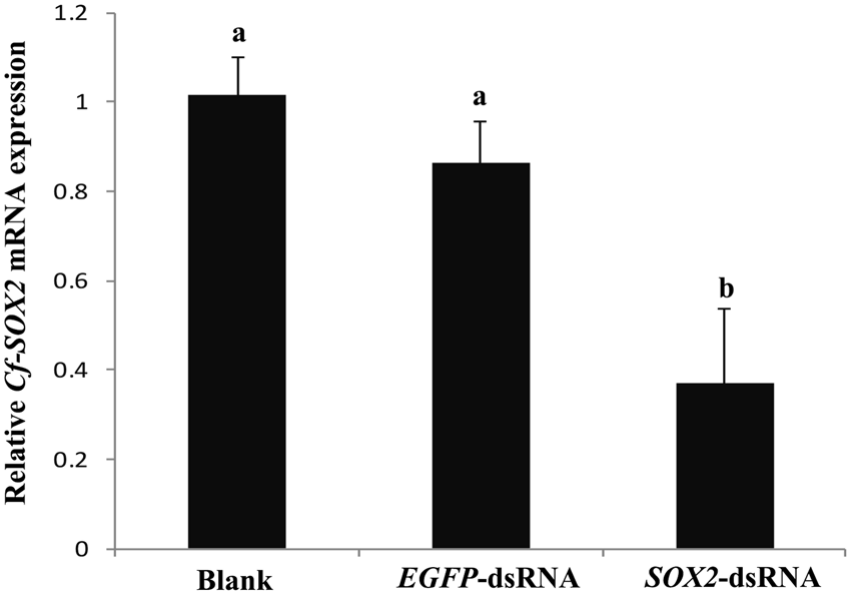
Level of *Cf-SOX2* mRNA in *C*. *farreri* testes on 72 h post injection of RNAi detected by qRT-PCR. Values are the mean ± SEM, n = 5; The expression level in the testes of blank group is set as 1.00 to calibrate the relative expression of other groups. Different letters (a,b) indicate statistically significant differences (*P* < 0.05).

The efficiency of *Cf*-*SOX2* knockdown was examined by qRT-PCR (Fig. 3). The level of *Cf-SOX2* mRNA in the *SOX2*-dsRNA testes was significantly decreased (*P* < 0.05), which was only 35% of that in the blank group. No significant difference of *Cf-SOX2* mRNA level was detected between the blank group and *EGFP*-dsRNA group.

**Figure 4:**
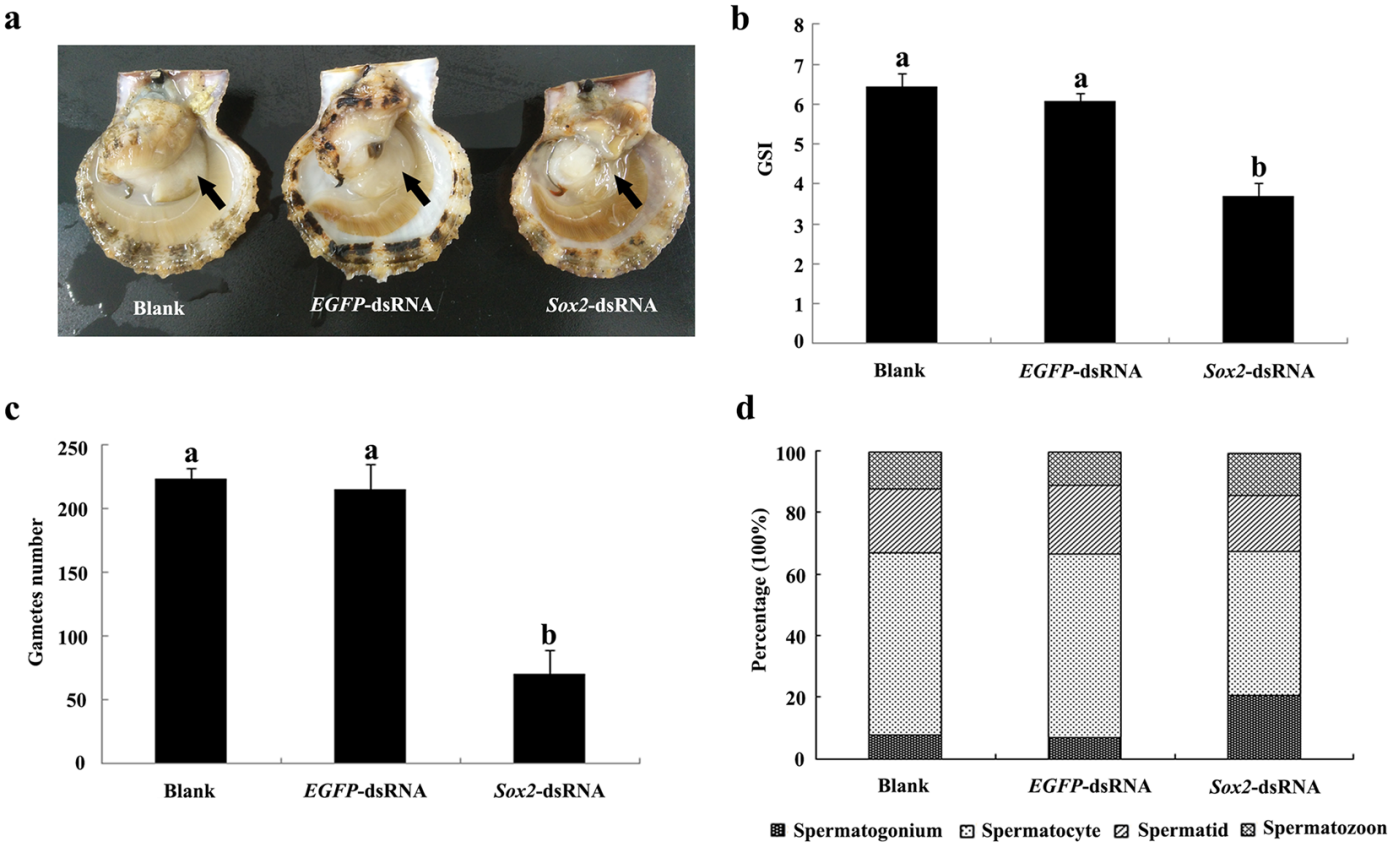
Characteristics of *C*. *farreri* testes after *Cf-SOX2* knockdown. (**a**) external morphology of *C*. *farreri* testes on the 21st day post RNAi; (**b**) GSI of *C*. *farreri* on the 21st day post RNAi (n = 6); (**c**) gametes number in testes of a 6400 μm^2^ area on the 3rd day post RNAi (n = 3, three sights of each sample, respectively); (**d**) percentage of various types of germ cells in a 6400 μm^2^ area on the 3rd day post RNAi (n = 3, three sights for each sample, respectively). Data are means ± SEM. Different letters indicate significant differences (*P* < 0.05).

The phenotype of the scallops presented obvious differences between the *SOX2*-dsRNA group and the control groups after *Cf-SOX2* mRNA was knocked down. In the *SOX2*-dsRNA injected group, the testes were translucent and wizened, and their size was much smaller than that of the control groups (Fig. 4a). The gonadosomatic index (GSI) of the *SOX2*-dsRNA injected scallops (3.68 ± 0.82) was significantly (*P* < 0.05) lower than that of the *EGFP*-dsRNA injected scallop (6.07 ± 0.52) or the blank scallops (6.45 ± 0.81) (Fig. 4b).

**Figure 5:**
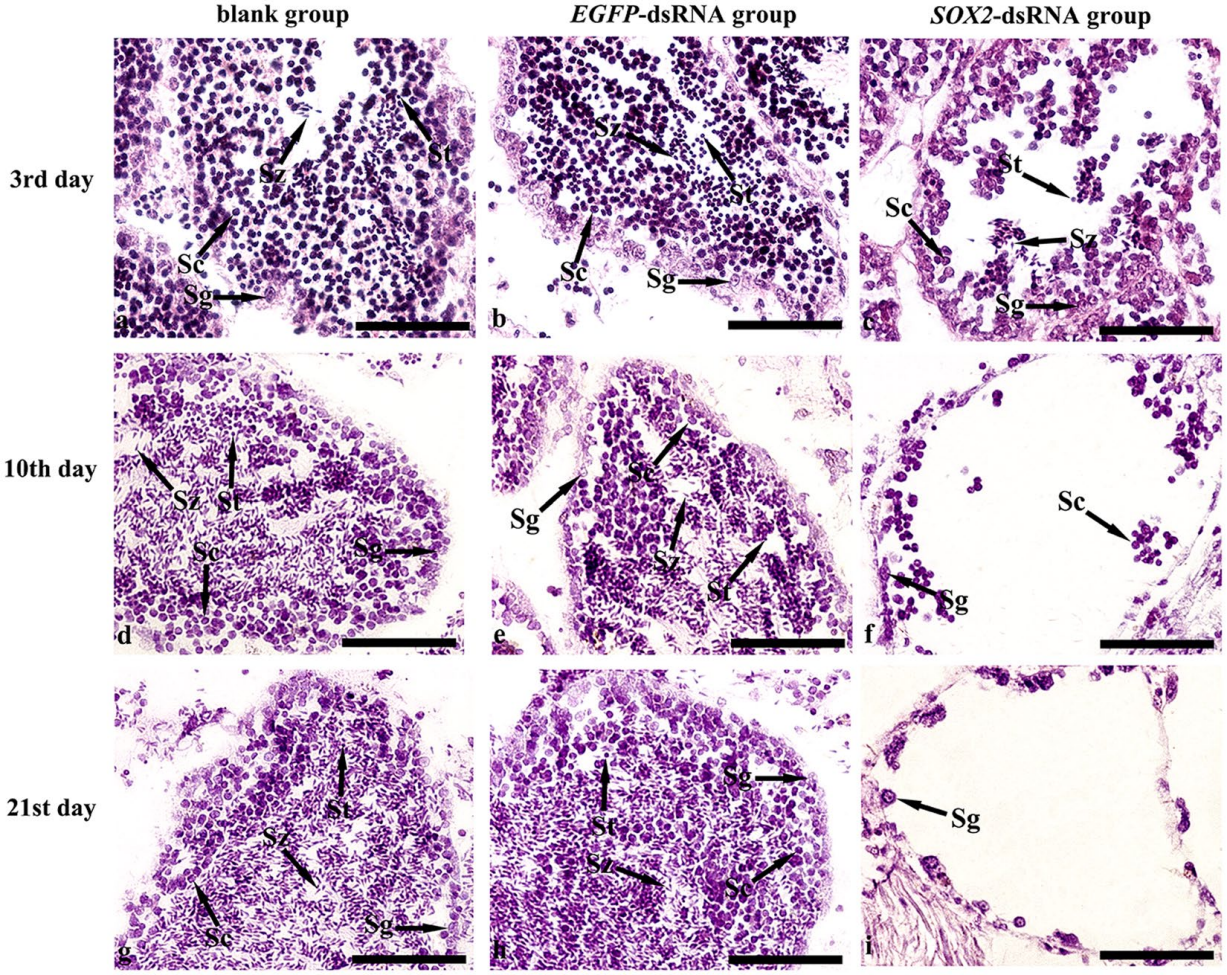
Histological observation of the *C*. *farreri* testes after the knockdown of *Cf-SOX2*. (**a**–**c**) Testes on the 3rd day post RNAi, the blank group (**a**), the *EGFP-*dsRNA group (**b**) and the *SOX2-*dsRNA group (**c**); (**d**–**f**), testes on the 10th day post RNAi, the blank group (**d**), the *EGFP-*dsRNA group (**e**) and the *SOX2-*dsRNA group (**f**); (**g**–**i**), testes on the 21st day post RNAi, the blank group (**g**), the *EGFP-*dsRNA group (**h**) and the *SOX2*-dsRNA group (**i**). Sg, spermatogonium; Sc, spermatocyte; St, spermatid; Sz, spermatozoon. Scale bar, 50 μm.

**Figure 6:**
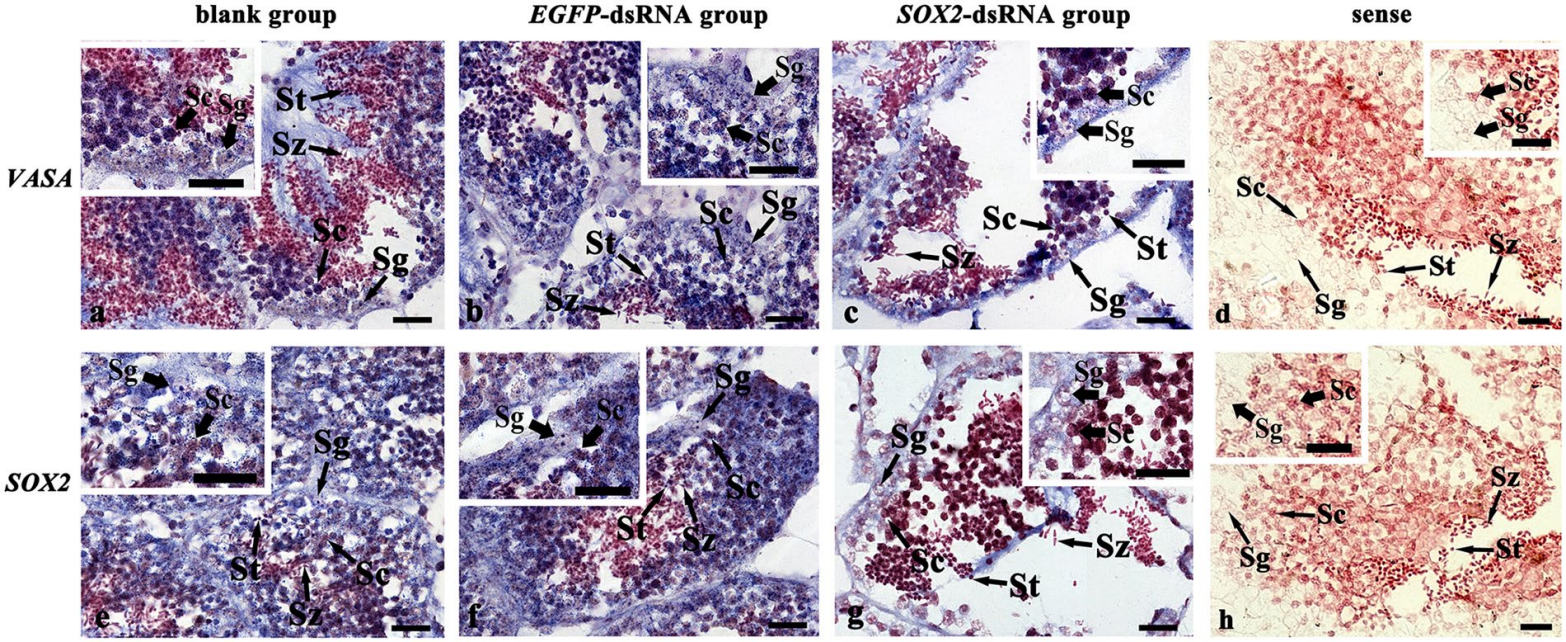
Status of germ cells in the *C*. *farreri* testes of 3 days post *Cf-SOX2* knockdown demonstrated by *in situ* hybridization. (**a**–**d**) Cyto-location of *Cf-VASA* mRNA; (**e–h**) cyto-location of *Cf-SOX2* mRNA. Positive signals with an antisense probe are indicated in blue (**a**–**c**,**e**–**g**). The negative control is conducted with sense probe (**d**,**h**). (**a**,**e**) The blank group; (**h**,**f**) the *EGFP-*dsRNA group; **c,g** the *SOX2-*dsRNA group. Sg, spermatogonium; Sc, spermatocyte; St, spermatid; Sz, spermatozoon. Scale bar, 10 μm.

The knockdown of *Cf-SOX2* expression level provoked defects of the germ cell development in testis (Fig. 5). On the 3rd day post injection, the germ cells in *SOX2*-dsRNA group loosely arranged in the germinal acini (Fig. 5c), and the number of the germ cells (70.0 ± 19.3 in 6400 μm^2^) reduced about 68% compared with the blank testis (223.0 ± 8.3 in 6400 μm^2^) which had no significant difference from that in *EGFP*-dsRNA testis (215.5 ± 19.6 in 6400 μm^2^) (Fig. 4c). Furthermore, cells in testes were labeled with *Cf-SOX2*, as well as the *C*. *farreri* germ cell marker gene *VASA* (*Cf-VASA*), through *in situ* hybridization, in order to identify different types of germ cells. The *Cf-VASA* transcripts were located in spermatogonia, spermatocytes, spermatids (Fig. 6a–c). While, in *SOX2*-dsRNA group, *Cf-SOX2* mRNA were weakly expressed in spermatogonia and few spermatocytes (Fig. 6g). No hybridization signal was detected in testes with the sense probe (Fig. 6d,h). On the 10th day post injection, only a few number of spermatogonia and spermatocytes loosely arranged in the germinal acini in *SOX2*-dsRNA group, while the germinal acini in control groups were filled with all kinds of germ cells (spermatogonia, spermatocytes, spermatids, spermatozoa) (Fig. 5d–f). When it is on the 21st day post injection, the germinal acini were nearly empty, and only several spermatogonia were observed in *SOX2*-dsRNA group (Fig. 5i). Moreover, the composition of germ cells was also changed on the 3rd day post injection. In *SOX2*-dsRNA testes, the germ cell composition was 20.8% spermatogonia, 47.2% spermatocytes, 18% spermatids and 14% spermatozoa, comparing to 8.0%, 59.2%, 20% and 21.1% of that in blank group which was similar in *EGFP-*dsRNA testes (Fig. 4d).

**Figure 7:**
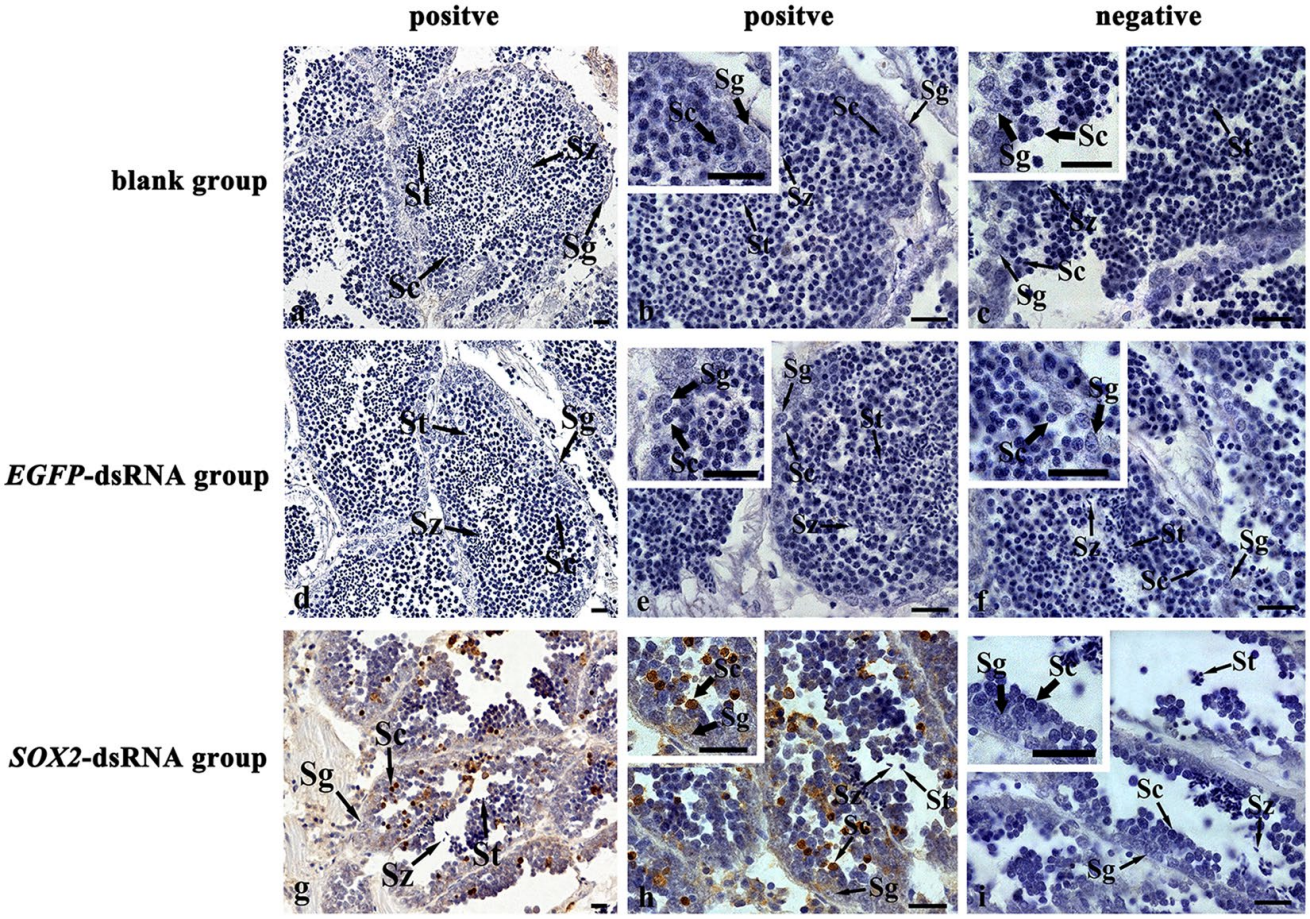
Cell apoptosis analysis by TUNEL in the *C*. *farreri* testes of 3 days post *Cf-SOX2* knockdown. Positive signals are indicated in brown. (**a**–**c**) The blank group; (**d**–**f**) the *EGFP*-dsRNA group; (**g–i**), the *SOX2*-dsRNA group; (**b**,**e**,**h**) magnified images of the gonads; (**c**,**f**,**i**) negative control without terminal deoxynucleotidyl transferase. Sg, spermatogonium; Sc, spermatocyte; St, spermatid; Sz, spermatozoon. Scale bar, 10 μm.

TUNEL detection revealed that many spermatocytes in *SOX2*-dsRNA testes after RNAi for 3 days presented obvious positive signals of apoptosis, while no obvious signals were visible in spermatogonia, spermatids and spermatozoa (Fig. 7g,h). Besides, the obvious positive signals were invisible in testes of *EGFP*-dsRNA group and the blank group (Fig. 7a,b,d,e).

### Transcriptome sequencing and *de novo* assembly

Transcriptome analysis were conducted from six testes of *C*. *farreri* in *SOX2*-dsRNA and blank groups (Table S1) and a total of 272,353,746 raw reads were generated (Table S2). After removing the adaptor sequences and ambiguous or low-quality reads, we obtained a total of 263,383,562 clean reads (96.70% of the raw reads), generating 131,340 assembled unigenes which had an average length of 1,149 bp and an N50 of 1,807 bp (Table S2, Fig. S1). All data have been submitted to the NCBI Sequence Read Archive (SRP125788).

### Functional annotation

**Figure 8:**
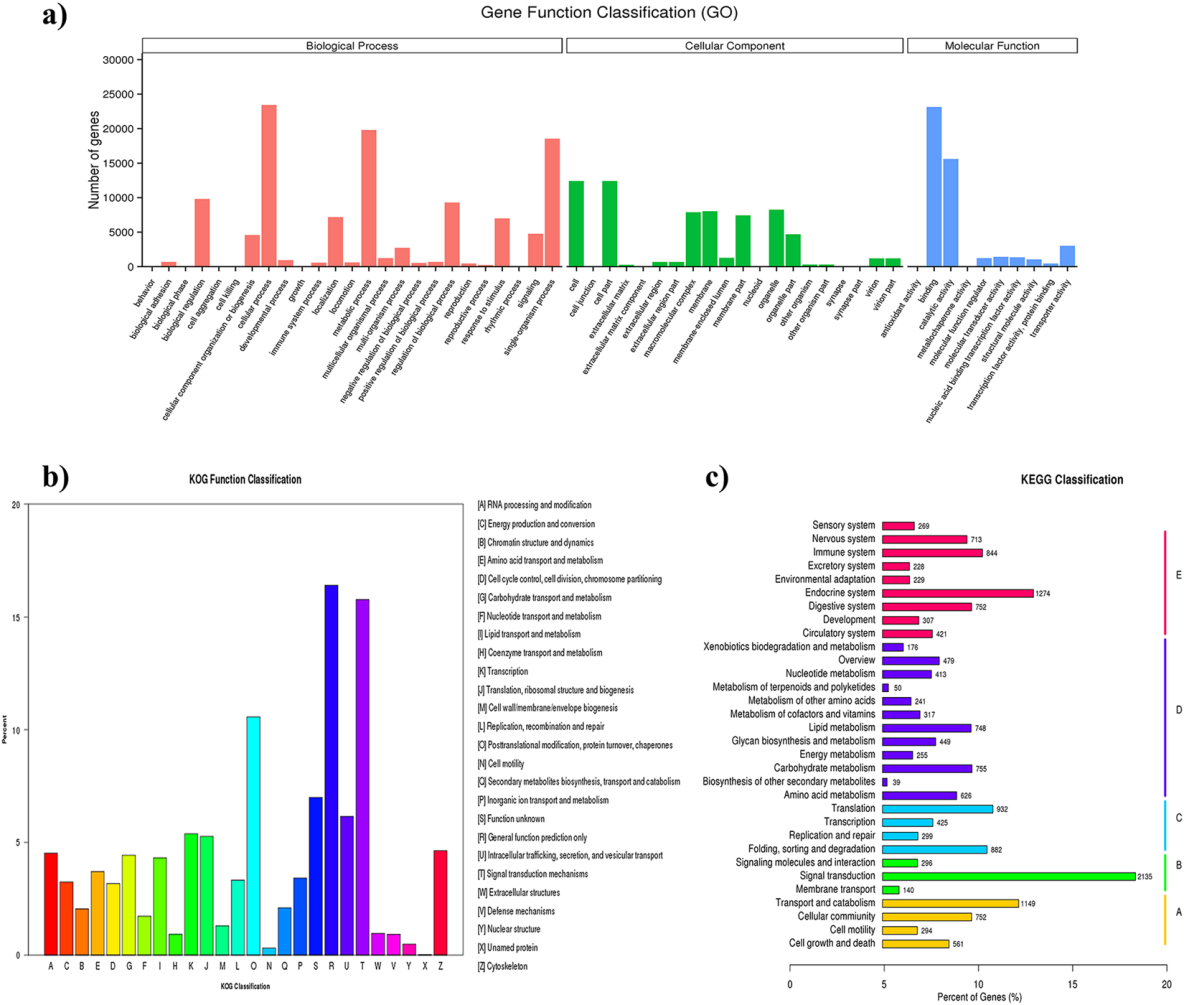
Function annotation of all unigenes in *C*. *farreri* testis. (**a**) Histogram presentation of the Gene Ontology (GO) classification; (**b**) Histogram presentation of clusters of eukaryotic Ortholog Groups (KOG) classification; (**c**) Histogram presentation of Kyoto Encyclopedia of Genes and Genomes (KEGG) classification. A, Cellular Processes; B, Environmental Information Processing; C, Genetic Information Processing; D, Metabolism; E, Organismal Systems.

Among the 131, 340 unigenes detected in the *C*. *farreri* testis, 56,396 (42.9%) were successfully annotated with at least one database (Table S2). According to the GO annotation, 41,327 unigenes were assigned to 55 level-2 GO terms under the three main GO categories, biological process, cellular component and molecular function (Fig. 8a). Cellular process (23441, 56.7%), metabolic process (19791, 47.9%) and single-organism process (18526, 44.8%) were the three most highly represented GO terms within the biological process, whereas binding (23129, 56.0%) and catalytic activity (15616, 37.8%) were the two most represented terms in molecular function.

Orthologous gene products were classified with the KOG database. 17,186 unigenes were allocated to 26 KOG classifications (Fig. 8b), among which “general function prediction only” (2820, 16.4%) was the largest group, followed by “signal transduction mechanisms” (2711, 15.8%) and “posttranslational modification, protein turnover, chaperones” (1816, 10.6%).

KEGG analysis assigned 15,901 unigenes to specific categories (Fig. 8c). Most of them fell into “organismal system” (5037, 31.7%), followed by “metabolism” (4548, 28.6%), “cellular processes” (2756, 17.3%), “environmental information processing” (2571, 16.2%) and “genetic information processing” (2538, 16.0%). The top five KEGG pathway were “signal transduction” (2135, 13.4%), “endocrine system” (1274, 8.0%), “transport and catabolism” (1149, 7.2%), “translation” (932, 5.9%) and “folding, sorting and degradation” (882, 5.5%).

### Differential expression analysis

**Figure 9:**
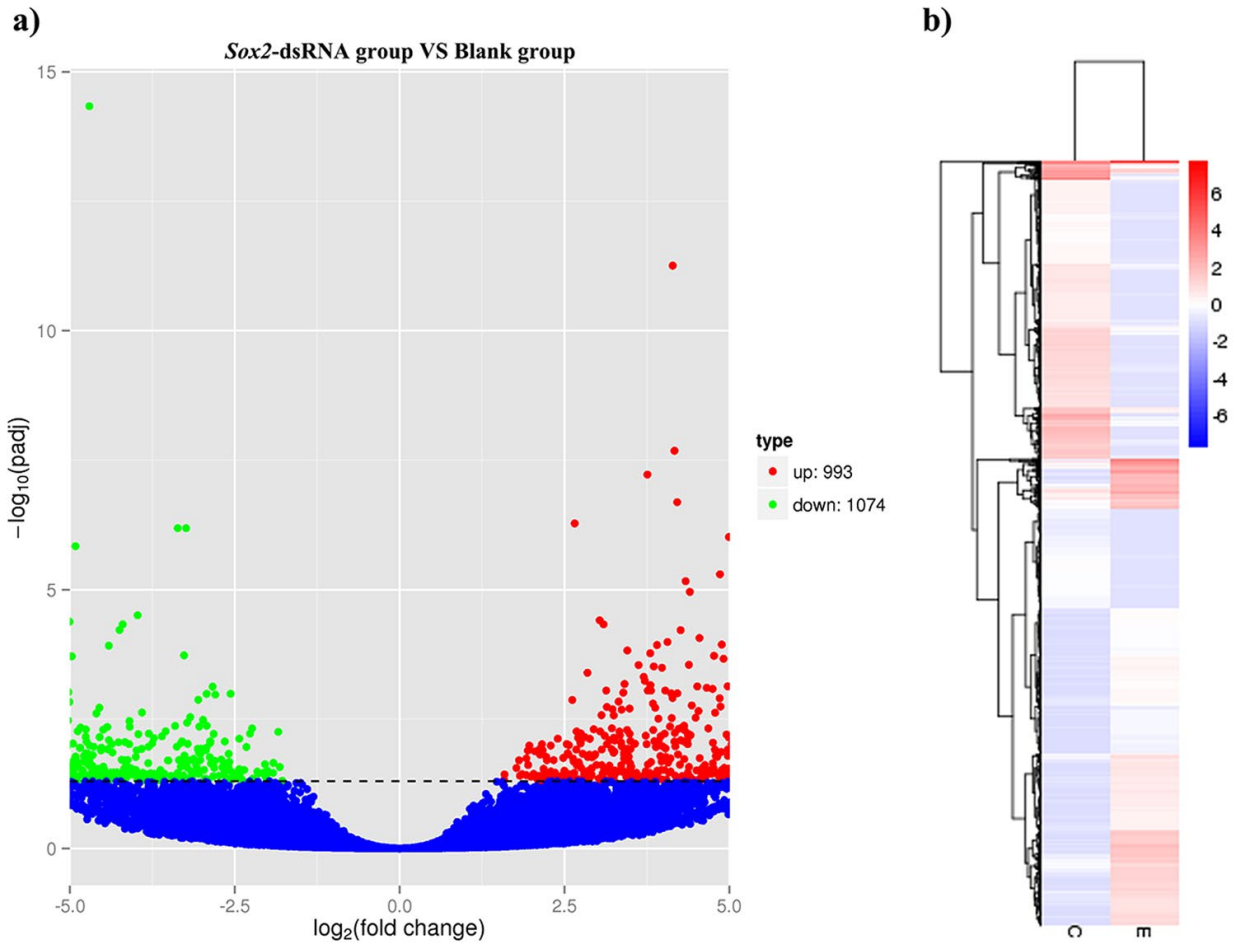
Analysis of differential expressed genes in the testes of *C*. *farreri* between *SOX2*-dsRNA group and blank group. (**a**) The volcano plot of DGEs. Red spots indicate significantly up-regulated genes; green spots indicate significantly down-regulated genes; blue spots are genes of no different expression. The significant difference is set at FDR < 0.05 to identify the different expression genes between two groups; (**b**) Clustering analysis of DEGs. Each column represents a group, and each row represents a gene. Red represents high level of expression, and blue represents low level of expression; numbers represent expression abundance. C, blank group; E, *SOX2*-dsRNA group.

A total of 2,067 DEGs were obtained between the *SOX2*-dsRNA group and bland group with FDR < 0.05 (Table S3), of which 993 (48.0%) were up-regulated and 1,074 (52.0%) were down-regulated after the knockdown of *Cf-SOX2* (Fig. 9a). Clustering analysis showed that the expression patterns of DEGs were obviously different between the blank group and *SOX2*-dsRNA group (Figs. 9b), and 6 subclusters could be generated (Fig. S2).

Based on GO annotation, a total of 38 DEGs were identified to involve in apoptosis, cell proliferation and reproduction (Table S3), such as regulation of apoptotic process (*casp2*, *casp3*, *casp8*), cell proliferation and growth (*bmp7*, *samd9*, *crebzf*, *iqsec1*), reproductive (*htt*, *tusc3*), mitotic cell cycle, meiotic cell cycle (*tle4*, *rprd2*) and spermatogenesis (*zmynd10*, *nipbl*, *mfge8*, *zfand3* and *armc3*).

KEGG pathway enrichment analysis showed that DEGs were enriched in 180 pathways. Among these pathways, 5 pathways enriched more than 5 DEGs, including cytosolic DNA-sensing pathway (6), spliceosome (10), viral carcinogenesis (9), herpes simplex infection (7) and influenza A (7). Moreover, the DEGs related to apoptosis and anti-apoptosis genes, such as *casp3*, *casp8*, *birc3* were enriched in apoptosis pathway, p53 signaling pathway and TNF signaling pathway (Table S4).

### Quantitative RT-PCR validation

**Figure 10:**
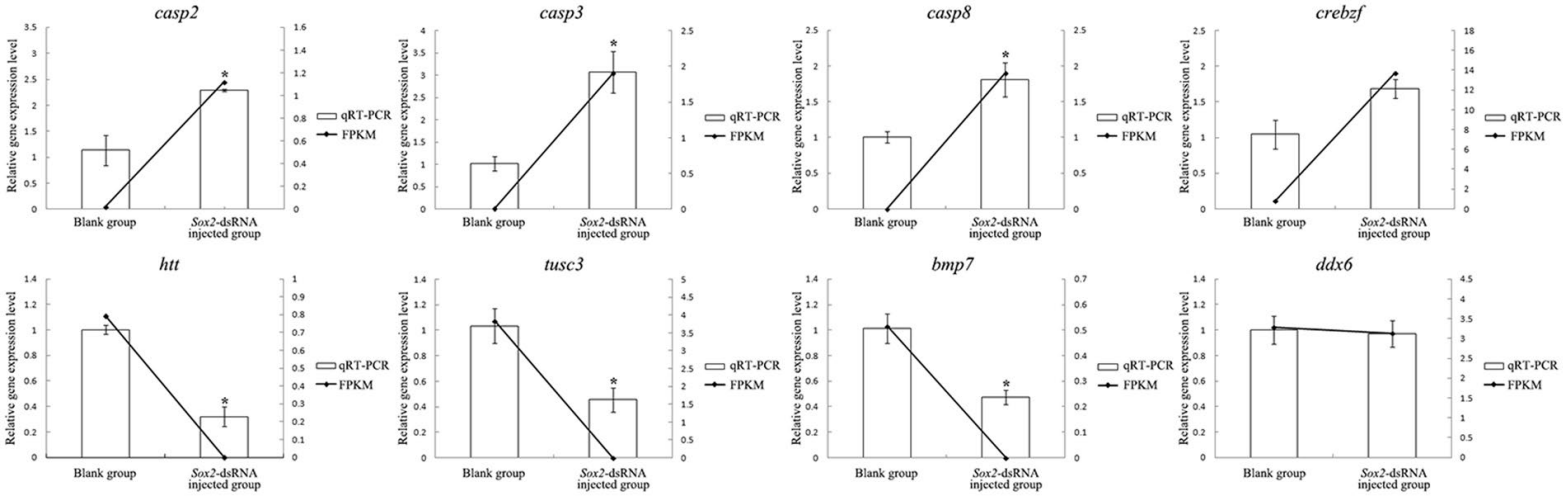
Validation of the RNA-Seq data of *C*. *farreri* testes using qRT-PCR. Vertical bars represent the qRT-PCR results; lines represent the FPKM values obtained from RNA-Seq analysis. Values are the mean ± SEM, n = 3; The expression level in the testes of blank group is set as 1.00 to calibrate the relative expression. Star (*) indicate statistically significant differences (*P* < 0.05).

The expression profiles of genes identified in RNA-Seq analysis were confirmed by detecting the relative mRNA levels of the following 8 genes by qRT-PCR: *casp2*, *casp3*, *casp8*, *crebzf*, *htt*, *tusc3*, *bmp7*, *ddx6* (Fig. 10). The results indicated that the data from qRT-PCR were basically consistent with those of RNA-Seq, and although the *crebzf* gene was not statistically significantly different in the qRT-PCR assay, it still showed the same differential expression tendency as revealed by RNA-Seq. The overall consistency confirmed the reliability of RNA-Seq data to accurately quantify the gene expression.

## Discussion

It has been reported that *SOX2* gene participates in the proliferation of PGCs and other processes during embryo development^2,5,11,12^. However, the studies of *SOX2* in mature gonad, especially in testis, are still limited, thus its function or participant biological processes are largely unclear. In this study, *Cf-sox2* mRNA was expressed significantly and located in all germ cells during reproductive cycle of testis. The joint study of experimental and bioinformatics analysis revealed that *Cf-SOX2* gene is essential to spermatogenesis and functional maintain in adult *C*. *farreri* testis. Our results will assist in better understanding of the regulatory mechanisms of *SOX2* gene in bivalve testis.

### *Cf-SOX2* regulates proliferation and apoptosis of spermatogonia and spermatocytes in *C*. *farreri*

In *M*. *musculus*, SOX2 protein are expressed in primordial germ cells (PGCs) of mouse embryo at 7.5 days post coitum (dpc), and involves in the proliferation of PGCs and the formation of spermatogonia. Campolo *et al*.^12^ found that in mouse embryos with *SOX2* conditionally knocked out by Cre/loxP, the number of PGCs is strongly reduced and spermatogonia in testes are completely depleted at 17.5 dpc. TUNEL and BrdU incorporation experiments show that the reduction of PGCs is mainly due to the decreased DNA synthesis rather than the increased apoptosis. Furthermore, proliferation of the cultured PGCs have also been verified to be associated with *SOX2*, with the fact that normal PGCs isolated from mouse embryo 8.5–11.5 dpc can proliferate *in vitro*, while those from the *SOX2*-knockout embryo do not.

In this study, the *Cf-SOX2* transcripts were revealed to locate in all germ cells of *C*. *farrier* testis, although its abundance in spermatocytes was slightly higher than that in spermatogonia, spermatids and spermatozoa (Fig. 2a,b,d–h). Moreover, the number of all spermatogenic cells dramatically reduced (Fig. 4c, 5a-c, 6) in testes 3 days post the knockdown of *Cf-SOX2*, and then only few spermatogonia were observable (Fig. 5g–i) until the 21st day. Therefore, we suggested that *SOX2* is involved in animal spermatogenesis besides regulating the proliferation of PGCs.

To explore the reason for the reduction of the spermatogenic cells, we carried out the TUNEL assay and found that obvious apoptosis was presented in spermatocytes of the *SOX2*-dsRNA testes (Fig. 7g,h). The differences between what we found in scallop and that in mouse implied *SOX2* may involve in different mechanisms of regulating the differentiation of spermatogenic cells at different stages. Furthermore, we found the number of spermatogonia in *SOX2*-dsRNA testes declined gradually with the progress of RNAi (Fig. 5c,f,i), while no TUNEL positive signal was observed. Based on the fact above, we proposed that the significant reduction of germ cell number might be resulted from the apoptosis of spermatocytes as well as the disability of spermatogonia proliferation and differentiation, and then lead to the failure of spermatid and spermatozoon formation.

### *Cf-SOX2* regulates spermatogenesis and testis functional maintenance through several biological pathways in *C*. *farreri*

Based on the transcriptome analysis performed with the testes 3 days post RNAi, a total of 2,067 DEGs were obtained between *Cf-SOX2* knockdown testis and normal testis. Functional and enrichment analysis showed that lots of DEGs and pathways have involved into the spermatogenesis and testis functional maintenance.

Some DEGs (*samd9*, *crebzf*, *iqsec1*, *bmp7*) involved in cell proliferation, mitosis and meiosis presented differential expression after *Cf-SOX2* knockdown. *samd9* is found to be related to cell proliferation and apoptosis. Its over expression in the colon cell line reduced the cell proliferation and boosted the CASP3 activity, while the proliferation rate restored after its knockdown with RNAi^31^. Analogous, the up-regulation of *crebzf* and *iqsec1* result in the inhibition of cell proliferation in mouse and human cells, and the down-regulation of *bmp7* result in the weaken of the proliferation ability^32–35^. In the present study, along with the testis retardance after *Cf-SOX2* knockdown, these four genes presented either up- or down-regulated expression patterns and based on their coincidence with the reported studies, we speculated *Cf-SOX2* could regulate the spermatogenesis through control the proliferation of germ cells.

Cell apoptosis is a process of programmed cell death, which controlled by a series of genes involving in the activation, expression and regulation processes. As the members of CASP family, CASP2 and CASP8 are apoptotic promoters, and CASP3 is the apoptotic executor, which plays an important role in cell apoptosis process^36–41^. cIAP2, encoded by *birc3*, is one of the members of IAPs (inhibitor of apoptotic proteins) family, which can block the apoptosis process by inhibiting the enzymatic activity of CASP3, CASP7 and CASP9^42–44^. In this study, a variety of genes (*casp2*, *casp3* and *casp8*) involved in cell apoptosis were up-regulated in testes after RNAi. Taking the results of TUNEL detection into consideration (Fig. 7g,h), we suggested that the activation of CASP apoptosis pathway in cells might be one of the reasons that resulted in the significant reduction of germ cells. Interestingly, the anti-apoptosis gene (*birc3*) was also up-regulated on the 3rd day of RNAi. We speculated it might be a stress response to cell apoptosis at the early stage.

Spermatogenesis is a process involving cellular proliferation, meiosis, and terminal differentiation that occurs during development of the testis and lots of specific genes involve in this process. *zmynd10* and *armc3* are important genes playing roles in sperm motility, and their deficiency cause sterility due to immotile sperm^45–48^. NIPBL is required for the association of cohesion with DNA and thus proper chromosome segregation. It is an important factor for mammals during early stages of meiotic prophase^49^. SED1, also known as MFG-E8, is expressed on the surface of sperm plasma membrane overlying the intact acrosome in mice and human, and plays an important role in sperm-egg binding^50–54^, while *tusc3* is expressed in spermatocytes^55^, and they have been suggested to be involved in male germ cell maturation. *Htt* mRNA is located in both primary and secondary spermatocytes, and the knockout of *htt* leads to significant decrease of spermatocytes and spermatids^56,57^. In the present study, all these genes were down-regulated after the knockdown of *Cf-SOX2*, which indicated *Cf-SOX2* could regulate the spermatogenesis process through these potential important downstream genes.

According to the KEGG pathway enrichment analysis, some pathways related to apoptosis, such as p53 signaling pathway, TNF signaling pathway and Apoptosis pathway, were enriched. P53 is a tumor suppressor protein that regulates the expression of a wide variety of genes involved in apoptosis, growth arrest, inhibition of cell cycle progression^58^. Tumor necrosis factor (TNF) also known as TNF-α is a pleiotropic cytokine and exerts its biological functions by interactions with two members of the TNF receptor (TNFR)^59^, both of which trigger several signal transduction pathways, including apoptosis mediated by CASPASE family^60,61^, the activation of NF-κB and JNK mediated by scaffolding protein TRAF (TNF receptor associated factors)^62^. Apoptosis, also known as programmed cell death (PCD), is the process of the autonomic death of pathological cell strictly followed by procedure in multicellular organisms, and is regulated by several genes. Among these pathways, the apoptotic genes such as *casp3*, *casp8*, *tp53i3* and cell cycle inhibiting gene *gadd45a* were differentially expressed after the knockdown of *Cf-SOX2*. Combining the TUNEL results in our research, we suggest that the DEGs of apoptotic related pathways may be regulated by *Cf-SOX2*, which together participate in germ cell apoptosis.

By means of transcriptome analysis, we suggested that the reduction of germ cells in testes after the knockdown of *Cf*-*SOX*2 could be resulted from the suppression of cell proliferation, the abnormal expression of spermatogenesis genes and the activation of apoptosis pathways. Several potential downstream genes of *Cf*-*SOX*2 were proposed and the subsequent expression characteristics and functional analysis of these genes could be studied in future for revealing detailed regulation mechanisms. Previous study of *KLF4* gene in *C*. *farreri* indicated that *Cf-KLF4* regulates early spermatogenesis, and promotes the differentiation of spermatogonia and spermatocyte, as well as the apoptosis of spermatocytes^23^. And our further transcriptomic study after *Cf-KLF4* was knockdown showed that several genes exhibited similar expression patterns (*IAPs*, *TNFR*, *TRAF* etc.) (unpublished data) as in this study, implying there may exist some correlation between *Cf-SOX2* and *Cf-KLF4* in testis development. Further studies are needed to explore the interrelation between these two genes, as well as the regulatory mechanism of spermatogenesis.

In the present work, *Cf-SOX2* mRNAs were located in spermatogonia, spermatocytes, spermatids and spermatozoa. After knockdown of *Cf-SOX2* mRNA in testes, the number of germ cells greatly reduced and a lot of spermatocytes went into apoptosis status, which lead to spermatogenic failure and indicated that *Cf-SOX2* played an important role in the development of testis and spermatogenesis. 2,067 DEGs were obtained through transcriptome analysis after the knockdown of *Cf-SOX2*, and among those DEGs, genes related to the spermatogenesis (*ZMYND10*, *NIPBL*, *MFGE8*, *ZFAND3*, *ARMC3*, *HTT*, *TUSC3*) were down-regulated, while other genes (*CASP2*, *CASP3*, *CASP8*, *BIRC3*, *SAMD9*, *CREBZF*, *IQSEC1*, *BMP7*) involved in cell apoptosis or cell proliferation were up- or down-regulated, indicating that *Cf-SOX2* regulates the development of spermatogenic cells and maintains the testis function through multiple biological pathways. Our study provides clues for exploring the regulatory mechanism of *SOX2* gene in bivalve testis development.

## Materials and methods

### Ethics statement

This study was approved by the Animal Ethics Committee of Ocean University of China, Qingdao, China.

### Experimental animals

Healthy male and female scallops *C*. *farreri* with mean shell height 6.19 ± 0.35 cm were collected from Shazikou Bay (Qingdao, China). The testes were excised, weighted and dissected, part of them were firstly fixed in 4% paraformaldehyde (dissolved in with 0.01 M phosphate buffered saline, PBS) at 4 °C for 24 h, then dehydrated with serial methanol (25%, 50%, 75%, 100%) diluted with 0.01 M PBS and stored in 100% methanol at −20 °C for histology and *in situ* hybridization. The remainders were immediately frozen in liquid nitrogen and stored at −80 °C for total RNA extraction.

According to histological characteristics and the gonadosomatic index (GSI = gonad weight/soft tissue weight × 100) described by Liao *et al*.^63^ and Liu *et al*.^64^, the testes of *C*. *farreri* used in our study were grouped into three stages, the proliferative stage (GSI = 4.07 ± 0.72), the growing stage (GSI = 6.90 ± 0.58) and the mature stage (GSI = 9.76 ± 1.46).

### Histology

Samples were immersed into 100% ethanol twice, cleared in xylene and embedded in paraffin wax. The 5-μm-thick sections were produced according to the procedure described by Liu *et al*.^64^. The sections were observed and photographed using a Nikon E80i microscope (Nikon, Tokyo, Japan).

### RNA extraction

Total RNAs were extracted using the conventional thiocyanate-phenol-chloroform method^65^, and were then incubated with DNase I (Takara, Dalian, China) to remove genomic DNA contamination, and purified using the RNeasy mini kit (Qiagen, Hilden, Germany) according to the manufacturer’s instruction. The RNA quality and quantity were assessed with electrophoresis and spectrophotometry.

### Quantitative RT-PCR (qRT-PCR)

qRT-PCR analysis was performed to determine the expression level of *Cf-SOX2* mRNA in testes at different stages. Based on the full-length sequence of *Cf-SOX2* (GenBank Acc. No. KF836755.1), two specific primers (Table 1) amplifying a 135 bp product were designed. The *C*. *farreri* elongation factor 1 alpha (*ef-1α*) (GenBank Acc. No. JQ278034.1) was selected as a reference gene^66^. The amplification was carried out in a 20 μl volume using Roche LightCycler 480 Real-Time PCR System (Roche, Basel, Switzerland) with SYBR Green Master Mix (Takara, Dalian, China) following the manufacturer’s instruction. Data were analyzed using the Roche LightCycler 480 system software version 1.5 (Roche, Basel, Switzerland), and the 2^−ΔΔCt^ method was used to analyze the relative levels of *SOX2* mRNA. All data were presented as mean ± SEM from five samples with three parallel repetitions. Besides, all qRT-PCR assays were validated in compliance with “the MIQE guidelines”^67^.

**Table 1:** List of primers used for qRT-PCR validation of RNA-Seq data of *C*. *farreri* testes.

Gene name	Primer sequence (5′-3′)	Amplificon length (bp)
*Cf-SOX2*	F: TCTCTCGGGGTAGCGGTTTC	135
	R: ATGTTCGTTTGCTCGGGTG	
*CASP2*	F: ATCACTGCCTCGTCATCG	165
	R: GTGTCATCCAAACGCTCC	
*CASP3*	F: GTCGGACAGAAGCGGTTA	108
	R: CAGCCTGGGACAGTAGAG	
*CASP8*	F: CCCCTGATGATAATGCCGAGAC	243
	R: GATTTGCGGGTGGTGGAT	
*CREBZF*	F: TCAGGCAAAAATCAACCG	142
	R: TTCATCTTCCAACACTCG	
*HTT*	F: CACCACGATACACTTAGG	194
	R: CAGCACGCATTCAACAAC	
*TUSC3*	F: TAAGTGGGTCGGAGAAAG	99
	R: CCAATCAGAGACAAGAGCAGG	
*BMP7*	F: TGTGACGGGGAGTGTTCG	106
	R: GGCTCGGGGCTTTGTAGG	
*DDX6*	F: AGCCCTTGTGATAGTCCCC	125
	R: TGTCATCCTTTAGGTTGG	

### *In situ* hybridization (ISH)

The 550 bp DIG-labeled RNA sense and antisense probes of *Cf-SOX2* were generated according to Liang *et al*.^29^. The 827 bp *Cf-VASA* probes were generated with the *C*. *farreri VASA* gene cDNA sequence (GenBank acc. no. DQ452383) and two specific primers *VASA*-F: 5′-TAATACGACTCACTATAGGGAGGAAGGACACGAGAACACGCAG-3′ (T7 promoter underlined) and *VASA*-R: 5′- ATTTAGGTGACACTATAGAAGCGCAGTAATACACGAGGACAGTC-3′ (SP6 promoter underlined). The testes were dehydrated in ethanol, cleared in xylene, embedded in paraffin, and then cut into 5-μm-thick sections for the testes at proliferative stage and the growing stage, and 3-μm-thick sections for mature stage. Sections were fixed to a slide with 0.1% polylysine at 37 °C for 12 h. *In situ* hybridization was performed as described in Feng *et al*.^68^, with modifications of samples digested for 10 min at 37 °C with 2 μg/ml protease K and counter stained with 1% neutral red. Both the *Cf-SOX2* and *Cf-VASA* mRNA were detected for the testes of 3rd day scallops after RNAi, while other samples were detected with *Cf-SOX2* probe only. Positive and negative detections were performed using the antisense and sense probe, respectively. The sections were observed and photographed using a Nikon E80i microscope (Nikon, Tokyo, Japan).

### RNAi assay

#### dsRNA synthesis

The procedure was performed as described by Suzuki *et al*.^69^ and Yang *et al*.^23^ with some modifications. Two specific primers, RNAi-F: 5′-TAATACGACTCACTATAGGGAGAGGACAAATACGCTCTACCAGG-3′ (T7 promoter underlined) and RNAi-R: 5′-TAATACGACTCACTATAGGGAGCATATGAGTAAGCGGAACAG-3′ (T7 promoter underlined) were designed according to *Cf-SOX2* cDNA sequence to amplify a fragment of 506 bp. The two primers of *EGFP*-dsRNA were used as described by Hou *et al*.^70^ to amplify a fragment of 497 bp used as control. Gel-purified PCR products were transcribed *in vitro* using the T7 MEGAscript RNAi Kit (Ambion, Austin, USA) to synthesis double-stranded RNA (dsRNA). The phenol/chloroform-extracted and ethanol-precipitated dsRNAs were suspended in RNase-free 0.01 M PBS (pH 7.4). The integrity and quantity of the dsRNA was assayed with electrophoresis and spectrophotometry.

#### dsRNA administration and sampling

Healthy male scallops *C*. *farreri* with mean shell height 6.28 ± 0.28 cm were purchased from the Nanshan Aquatic Product Market (Qingdao, China). Seventy-five male scallops at the proliferative stage were evenly assigned and raised in three aquaria with 540 L filtered, aerated seawater at 16.0 ± 0.2 °C, respectively. The scallops were fed with unicellular algae *Isochrysis galbana* and *Chaetoceros muelleri* and water was renewed daily during the experiment. Eighty microliter PBS containing 30 μg *SOX2*-dsRNA (*SOX2*-dsRNA group) or 80 μl PBS containing 30 μg *EGFP*-dsRNA (*EGFP*-dsRNA group) were injected into the adductor muscle of male scallops at T0 (initiation of this assay) and T7 (the 7th day), respectively. Both the *EGFP*-dsRNA and the blank (no injection) groups were used as controls. The testes were weighted and sampled at 3rd day (five scallops for each group), 10th day (three scallops for each group) and 21st day (six scallops for each group) post RNAi, respectively.

#### Detection of histological feature after RNAi

Testis sections of the sampled scallops from the blank group, *SOX2-dsRNA* group and *EGFP-dsRNA* group were made following the method in Section “Histology**”**. The tissue characteristics of testes and the germ cell types were observed with Nikon E80i microscope (Nikon, Tokyo, Japan).

#### Quantification and composition of germ cells in the follicles of testes

Testis sections of the scallops from 3rd day were inspected under microscope to determine the quantification and composition of germ cells. All germ cells in the follicles of testes were distinguished according to Liu *et al*.^64^ and counted by randomly observing three sights (6400 μm^2^ for each) in histological sections of testes, and percentage of germ cells at different groups was calculated.

#### TdT-mediated dUTP Nick-End Labeling (TUNEL) assay

By using a DeadEnd^TM^ Colorimetric TUNEL System Kit (Promega, Madison, USA), the TUNEL assay was conducted to detect cell apoptosis in the testes of 3rd day scallops according to the manufacturer’s instruction. Sections of the testis were prepared following the method in Section “Histology”. Observation and digital images were taken with Nikon E80i microscope (Nikon, Tokyo, Japan).

### Transcriptome analysis

#### Sample preparation and library construction for transcriptome sequencing

The testes of three scallops on the 3rd day post RNAi and from each of *SOX2*-dsRNA group and blank group were used for transcriptome sequencing. The method of total RNA extraction was the same as Section “RNA extraction”.

A total amount of 1.5 µg RNA per sample was used for library preparation. Sequencing libraries were generated using NEBNext^®^ Ultra™ RNA Library Prep Kit for Illumina^®^ (NEB, Ipswich, USA) following manufacturer’s recommendations. The libraries were subjected to an Illumina Hiseq. 2000 platform with pair-end sequencing of 150 bp.

#### *De novo* transcriptome assembly and functional annotation

Raw reads were first preprocessed for quality control to obtain the clean reads by removing reads containing adapters, ambiguous ‘N’ nucleotides (with the ratio of ‘N’ to be more than 10%), and low quality reads (with the Phred quality scores ≤ 20). Then all clean reads from the six libraries were jointly assembled using Trinity software as described for *de novo* transcriptome assembly^71^. Unigenes were aligned with diamond and NCBI blast 2.2.28+ to the databases NR, NT and Swiss-Prot with an E-value threshold of 1.0E-5, as well as KOG with an E-value threshold of 1.0E-3 to predict the function of genes. Blast2GO^72^ software was used to obtain GO annotations. Pathway assignments were determined based on the KEGG database using KAAS (KEGG Automatic Annotation Server, http://www.genome.jp/kegg/kaas/) with an E-value threshold of 1.0E-6.

#### Differential expression analysis

Gene expression levels for each sample were estimated using RSEM^73^ and each unigene was normalized by fragments per kilobase of exon model per million mapped reads (FPKM)^74^. Differential expression analysis of these two groups was performed using the DESeq R package (1.10.1)^75^. The resulting *P* values were adjusted using the Benjamini-Hochberg’s false discovery rate (FRD)^76^. Genes with an FDR < 0.05 found by DESeq were assigned as differentially expressed.

#### GO and KEGG enrichment analysis

Gene Ontology (GO) enrichment analysis of the differentially expressed genes (DEGs) was implemented by the GOseq R packages based Wallenius non-central hyper-geometric distribution^77^. KOBAS^78^ software was utilized to test the statistical enrichment of differential expression genes in KEGG pathways.

#### qRT-PCR validation

Expression levels of 8 selected DEGs, *casp2*, *casp3*, *casp8*, *crebzf*, *htt*, *tusc3*, *bmp7*, *ddx6* were validated using qRT-PCR with the 6 RNA samples of testes used for the transcriptional analysis. Gene-specific primers (Table 1) were designed using Primer 3 (http://primer3.ut.ee/) based on their ORF sequences. All procedures were identical as in Section “Quantitative RT-PCR (qRT-PCR)**”**.

### Statistical analysis

All data were presented as means ± SEM. Significant differences between means were tested using one-way analysis of variance (ANOVA) followed by Tukey’s HSD test (SPSS software version 18.0; SPSS, Chicago, USA), and the significant level was set at *P* < 0.05.

## Electronic supplementary material

Dataset1

## Data Availability

Raw sequence reads of RNA-seq can be found in the NCBI Sequence Read Archive (SRP125788).
